# Genomic and Physiological Basis of Structural and Foliar Trait Variation in Tropical Species *Pterocarpus officinalis* : Implications for Restoration in Future Drier Climates

**DOI:** 10.1111/eva.70102

**Published:** 2025-04-28

**Authors:** Sean M. Collins, Kevin C. Grady, Gustavo P. Lorenzana, Kailey Baesen, Laura Figueroa‐Corona, E. Petter Axelsson, Aalap Dixit, Amanda R. De La Torre

**Affiliations:** ^1^ School of Forestry Northern Arizona University Flagstaff Arizona USA; ^2^ Department of Wildlife, Fish and Environmental Studies Swedish University of Agricultural Sciences Umeå Sweden; ^3^ Department of Natural Resource Ecology and Management Oklahoma State University Stillwater Oklahoma USA

**Keywords:** GWAS, local adaptation, *Pterocarpus*, restoration, water use efficiency, whole‐genome sequencing

## Abstract

Tropical wetlands are some of the most threatened ecosystems in the world. *Pterocarpus officinalis* exists in swampy wetlands in riparian and fresh‐water coastal areas across the neotropics, supporting biodiversity and storm surge and flooding protection as well as water filtration. In Puerto Rico, *P. officinalis*‐dominated forests have been severely declining in recent decades, mainly due to land development. Reversing this trend in the face of climate change and projected sea level rise via ecological restoration may benefit from understanding phenotypic traits suitable for future climates. Currently, there are no seed sourcing guidelines for restoration, due to the understudied nature of the species. The goals of our study were to examine population structure and the genomic basis of variation in structural and physiological foliar traits. Seeds were collected from twelve seed sources spanning the natural distribution of *P. officinalis* in Puerto Rico. Water use efficiency related foliar traits were measured in well‐watered conditions from seedlings grown in a nursery experiment. A total of 109 seedlings were whole‐genome resequenced from 12 seed sources. Our results indicate strong foliar trait variation despite very little genetic differentiation among seed sources within the island, suggesting a relatively small number of genes might be involved in water‐use efficiency traits. Eleven out of thirteen foliar traits varied significantly across seed sources. Trait variation was associated with either longitude, elevation, mean annual precipitation, or isothermality. Seedlings across seed sources were observed to have different strategies for managing water use. Candidate loci identified using Genome‐Wide Association Studies were associated with signal transduction, transcription regulation, DNA and RNA methylation, transport, and primary and secondary metabolism. Restoration of this species is key in maintaining ecosystem services. Our study identified seed sources that may be successful in drier restoration conditions and match future arid climates.

## 1 Introduction

Rapidly changing climates along with land development across the world are leading to increased stress and habitat loss for many forest tree species (Pykälä 2019; Rands et al. 2010). Land development and climate change provide a twofold interaction as losing land to development lowers population size for a species, potentially leading to a loss of genetic diversity which can hinder the adaptive capability required for changing climates (Aguilar et al. 2008; Chung et al. 2023; Lowe et al. 2005; Young et al. 1996). Natural variation in climate combined with habitat loss may have already differentiated the phenotypic responses within forest tree species, leading to drastically different trait responses among different seed sources (Bonan 2008). Therefore, understanding the molecular mechanisms of phenotypic variation can help inform conservation efforts as inferences can be made on how certain populations may respond to future climate conditions. Restoration efforts can then select specific seed sources that are well suited to their new area based on genetic structure and specific phenotypic responses. This practice should lead to more successful restoration efforts by pre‐adapting forest restoration to future conditions (Axelsson et al. 2020).

Drought and heat events are predicted to increase in frequency and intensity as a consequence of climate change, bringing significant challenges to forest ecosystems. Water‐use efficiency (ratio of carbon gain to water loss) is an extraordinarily important process and plays a major role in the survival of all tropical plant species (Nandy and Ghose 2001; Clough and Sim 1989; Andrews and Muller 1985). Individuals that use water more efficiently are more likely to survive longer in drier areas and/or during drier periods. Water‐use efficiency traits, which include gas exchange, leaf morphology, and stable isotope discrimination, are subject to selection pressures (e.g., abiotic factors) that can lead to variation in phenotypes specific to each seed source (Rahman et al. 2019, 2020; Brienen et al. 2011). Variation in phenotypes across seed sources can play a major role in how seed sources perform in field settings. In the face of a projected drier future climate for many areas throughout the tropics (Colón‐Rivera et al. 2014; Larsen 2000; IPCC 2007), water‐use efficient seed sources may perform better in planting areas where higher water stress is expected. Identifying candidate loci for these water‐use traits can complement phenotype measurements, as this helps to elucidate molecular drivers of phenotypic variation. From there, seed sources can be chosen based on adaptive genes or other molecular mechanisms that are associated with water‐use profiles specific to each restoration setting.

The Dragonsblood Tree ( *Pterocarpus officinalis* ) is an understudied and declining mangrove associate species that is in great need of restoration. *P. officinalis* is present in swampy‐wetland forests in riparian and coastal areas across the Caribbean and can heavily dominate stands in freshwater coastal swamps (Eusse and Aide 1999; Imbert et al. 2000; Muller et al. 2006; Rivera‐Ocasio et al. 2002). *P. officinalis* supports a wide variety of biodiversity (Imbert et al. 2000; Mata et al. 2011) and can provide ecosystem services to many people, such as flooding prevention (Marois and Mitsch 2015), biodiversity support, and limiting soil erosion (Bâ and Rivera‐Ocasio 2015). However, these services are threatened as *P. officinalis* has been declining in recent years (Muller et al. 2009), with the IUCN listing it as Near Threatened across its range (Barstow and Klitgård 2018). The decline of *P. officinalis* has been especially severe within Puerto Rico (Muller et al. 2009; Cintrón 1983; Adams et al. 1999) due to land conversion for agriculture or urbanization. Within Puerto Rico, 14 stands are believed to remain, which translates to roughly 300 ha down from more than 10,000 ha (Adams et al. 1999). With the large decrease in *P. officinalis*, there is a great need for restoration to better preserve local biodiversity and increase the amount of ecosystem services provided to the public. Unfortunately, *P. officinalis* restoration efforts are currently hindered as there are currently no seed sourcing guidelines available due to the relatively understudied nature of the species.

Developing climate adaptive seed sourcing guidelines is particularly important due to the combination of predicted future stressors and *P. officinalis* water‐use strategies. Future drier climates and higher sea levels in the Caribbean will reduce the availability of freshwater inputs, leading to episodic drying events in wetlands (IPCC 2007; Gamble 2014; Gamble and Curtis 2008; Watts 1995). These episodic drying events and reduced freshwater inputs most likely will lead to increased water stress for Puerto Rican *P. officinalis* , as it primarily relies on surface water for freshwater inputs rather than groundwater (Colón‐Rivera et al. 2014; Larsen 2000). Therefore, identifying seed sources that could perform well in drier conditions and the alleles that drive this higher water‐use efficiency can greatly increase the chance of restoring these wetland forests in the face of changing climates (Colón‐Rivera et al. 2014; Larsen 2000; IPCC 2007). The small body of literature for Puerto Rican *P. officinalis* suggests strong variation in many other physiological and structural traits related to the effects of salinity, gas exchange rate, and foliar nutrient composition (Bompy et al. 2015; Colón‐Rivera et al. 2014; Munns 2002; Parida and Das 2005). Previous small‐scale genetic studies have shown evidence for four distinct genetic clusters of *P. officinalis* within Puerto Rico based on AFLP methods (Rivera‐Ocasio et al. 2006). Chloroplast and nuclear microsatellites suggest that Puerto Rican *P. officinalis* populations are distinct from populations in the rest of its range (Muller et al. 2009). The genomic basis of water‐use efficiency has never been examined in Puerto Rican *P. officinalis* .

The main goals of our study are (1) to assess the fine‐scale population structure of the species using genome‐wide molecular markers, (2) to characterize fine‐scale variation in physiological and structural foliar traits related to water‐use efficiency, (3) to detect candidate loci for water‐use efficiency traits through GWAS and GEA, and (4) to provide foundational guidelines for climate smart seed sourcing. The results of our study will be the first to provide a genome‐wide assessment of *P. officinalis* population structure across the island and assess potentially adaptive differences in water‐use efficiency among different seed sources. Our study will also add to the small body of literature for this species, all of which can go towards better informing seed sourcing guidelines and restoration efforts.

## 2 Material and Methods

### 2.1 Nursery Experiment and Sample Collection

**FIGURE 1:**
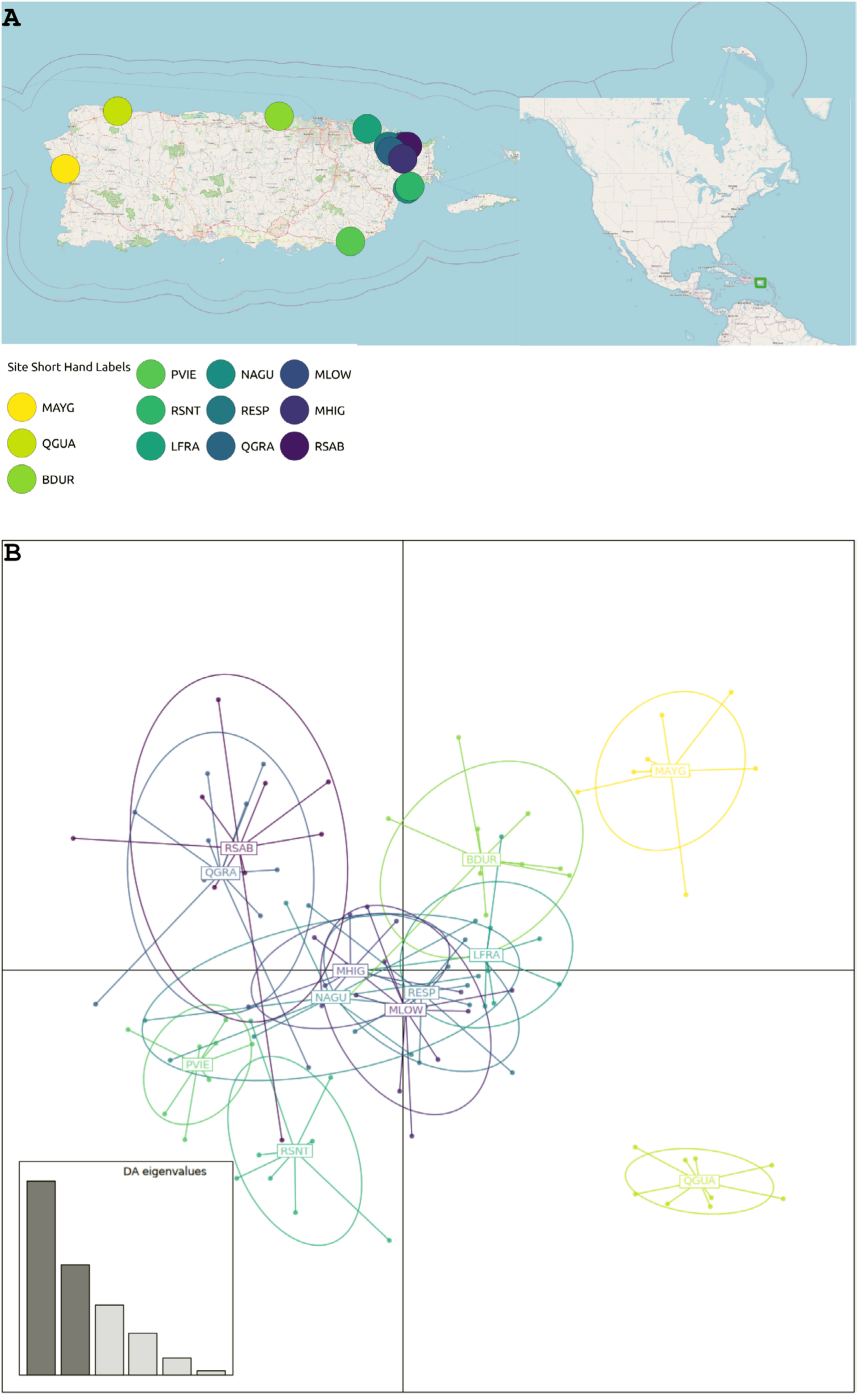
Collection map. Top (A), map of Puerto Rico with the inset of the southeastern US, Gulf Coast and Central America. Seed source locations used in this Study are shown in colored dots with the shorthand code found in Table 1. Red lines within island borders indicate major roads. Lines surrounding the island indicate the bounds of the Puerto Rican territory. Bottom (B) Discriminant Analysis of Principal Components Plot using the 17,563 SNP dataset samples are colored by seed source locations. Inset shows the percent variation accounted for by each discriminant axis.

To examine seed source variation in *P. officinalis* , we collected seeds from 9 randomly selected mother trees separated by at least 25 m from one another from each of 12 seed source locations distributed across 17°–18° North latitude and 65°–67° West longitude in Puerto Rico (Tables 1 and S2, Figure 1). We used these 12 seed source populations out of the total of 15 known populations in the entire island of Puerto Rico because they represented all viable (e.g., seed producing) populations remaining and were logistically permissible from which to collect seeds. In October 2020, seeds were germinated in a nursery in Cupey, Puerto Rico in 0.5‐gal polyvinyl containers using a soil medium consisting of a mixture of peat moss, vermiculite, and sand. Seedlings from all seed sources were produced under ambient environmental conditions present at the nursery plus supplemental watering. Seedlings were maintained at soil moisture field capacity throughout the production and measurement stages.

**TABLE 1:** Locations of seed sources used in our *P. officinalis* study.

Collection site	Collection site code	Latitude	Longitude	Elevation (ft)	Min temp of the coldest month	MAP	Isothermality	Precipitation of wettest quarter
Riego Los Frailes	LFRA	18.42	−65.91	9.84	19.70	1529	72.25	500
Rio Mameys	MLOW	18.35	−65.76	107.67	19.30	1967	69.64	629
Cubina Bosque Durado	BDUR	18.47	−66.28	9.84	18.90	1671	75.86	493
Rio Espiritu Santo	RESP	18.35	−65.82	140.82	19.10	1914	70.45	616
Rio Sabana/Jimenez	RSAB	18.35	−65.74	196.52	19.40	1931	69.88	622
Quebrada Jimenez/Laboy	QGRA	18.33	−65.81	837.6	18.30	2181	69.83	687
Naguabo	NAGU	18.18	−65.74	3.28	19.90	1795	71.38	642
Rio Santiago	RSNT	18.19	−65.73	3.28	19.90	1781	70.72	633
Rio Guajataca	QGUA	18.49	−66.96	3.48	17.30	1587	78.32	515
Mayagüez	MAYG	18.26	−67.18	3.28	17.80	1634	78.40	660
MHIG	MHIG	18.30	−65.76	887	17.70	2266	67.72	692
Punta Vientos	PVIE	17.97	−65.98	0	19.90	1493	72.32	618

*Note:* In‐text shorthand name used for each seed source are shown in Collection site code column. Lat, Long and a selection of climate variables are presented to highlight some of the environmental variation across the study area.

### 2.2 Whole‐Genome Re‐Sequencing

Leaf tissue was collected from nursery‐grown seedlings prior to outplanting in the common garden from 109 1‐year‐old seedlings in July 2021. Individuals sequenced per seed source ranged from 7–10. DNA was extracted using the E‐Z 96 Plant DNA DS kit (Omega Bio‐tek, Norcross, GA), and concentration was measured using a Qubit 4 Fluorometer (Thermo Fisher, MA, USA). After library preparation, libraries were sequenced in two lanes of an Illumina NovaSeq 6000 sequencer using 150 bp paired‐end reads. Library prep and sequencing were done at Rapid‐Genomics (LGC Biosearch Technologies, Gainesville, FL). An average of 9,360,098 sequence reads were generated per library. After adapter removal, quality filtering was performed with fastp (v0.23.2; Chen et al. 2018) to retain a total of 8,647,863 sequence reads with at least 140 bp in length and a minimum Phred‐scaled quality score of 30. Summary information for sequencing data is shown in Table S1. Contamination detection was performed using Kraken (v 2.1.1; Wood and Salzberg 2014) to produce curated and clean reads.

### 2.3 SNP Calling

High‐quality reads were mapped to the *Pterocarpus macrocarpus* genome (PRJNA1006136; Yang et al. 2023) using the BWA‐MEM algorithm (v0.7.10; Li and Durbin 2009) with a mean alignment rate across samples of 79.88%. Default parameters for BWA‐MEM were used. The *P. macrocarpus* genome was chosen as it was one of the few reference genomes available for the species in the *Pterocarpus* genus and gave the highest alignment rate for our reads compared to mapping to *Pterocarpus santalinus* . Alignments were converted to binary files using samtools (v1.5; Li et al. 2009) and unmapped reads were discarded. Single‐nucleotide polymorphisms (SNPs) were discovered and processed with FreeBayes software (v.1.2.0; Garrison and Marth 2012), a haplotype‐based caller under a Bayesian statistical framework. The *P. macrocarpus* genome was used as a reference and to map reads of each sample. Genotypes were then merged with vcftools (v0.1.16; Danecek et al. 2011) to get a total of 55,188,566 SNPs. The initial SNP set was filtered with vcftools (v0.1.16; Danecek et al. 2011) using the following parameters: (1) the allelic number was equal to 2; (2) minimum sequence depth was above 10; (3) quality mapping above 20 and (4) minor allele frequency (MAF) was greater than 0.05; and (5) indels were also removed to limit any confounding issues from mapping to a different species to obtain a final set of 56,700 SNPs. All the bioinformatic processing is documented at https://gitlab.com/lcorona/dragonsblood‐tree‐genomics.

### 2.4 Foliar Trait Measurements

Foliar traits were measured on eleven out of the twelve genotyped seed sources in June 2021. The seed source not sampled was MHIG (Table 1) due to a lack of adequate foliar tissue availability. We randomly sampled 10 seedlings for nine out of the eleven remaining seed sources. All available seedlings from the remaining two seed sources were included in the study (QUE‐BRA had 9 individuals sampled and BOS‐DUR had 7 seedlings sampled). Therefore, a total of 106 seedlings across 11 genotyped seed sources are included for foliar trait measurements.

A suite of morphological and physiological foliar traits that influence survival, growth, resilience, and performance of planted seedlings in restoration projects was measured for these 106 1‐year‐old seedlings in June and July 2021. The foliar traits selected are often correlated to adjustments that plants make to cope with varying degrees of water availability. Specifically, traits like specific leaf area (SLA), leaf thickness, and foliar nitrogen concentration (%N) are part of the leaf economics spectrum and provide insights on trade‐offs between resource acquisition and conservation strategies across seed sources. These traits, measured along with instantaneous leaf‐level gas exchange measurements such as transpiration rate (E), stomatal conductance (gs) and net photosynthetic rate (A) and traits developed over a longer timeframe such as carbon isotope discrimination (d13C), nitrogen discrimination (d15N), and foliar carbon concentration (%C) provide insights on seed source variations in balancing carbon gain and water loss (water use efficiency) while growing in a common environment.

In July 2021, Leaf‐level gas exchange was measured using a portable gas exchange measurement system (Li‐6800, LiCor, Lincoln, NE, USA) between 10:00 and 14:00 h on the most recently developed, fully formed leaves. All measurements were taken on well‐watered seedlings maintained at field capacity. We measured three seedlings per population each day to stratify sampling among populations to account for temporal variation. Within each population, gas exchange measurements each day were conducted evenly over hours to minimize confounding of measurement time and population. One seedling from each seed source was measured within each time block each day and repeated three times per day. A 6 cm^2^ foliar area was examined per leaf where this amount of area was placed in the LiCor chamber where gas fluxes are estimated. Conditions controlled inside the chamber were: relative humidity = 65%, CO_2_ = 420 ppm, temperature = 30°C, and photosynthetic photon flux density = 1500 μmol m^–2^ s^–1^. We measured net photosynthetic rate (A), stomatal conductance (gs), transpiration rate (E), and calculated instantaneous, intrinsic water‐use efficiency (A/gs).

Specific leaf area (SLA; leaf area/leaf mass) was measured on three randomly selected, most recently developed, fully formed leaves per seedling. The leaves were oven‐dried at 70°C for 48 h and then weighed. Fresh leaf thickness was measured between the midrib and the margin of two out of the three fully expanded leaf samples for SLA using a digital caliper on all seedlings. On these same seedlings, an index of leaf chlorophyll content (SPAD) was assessed using the SPAD‐502 chlorophyll meter. All SPAD measurements were conducted between the midrib and leaf margin.

In August 2021, three randomly selected, most recently developed, fully formed leaves per seedling (106 total seedlings) were sampled for measurement of carbon isotope discrimination, nitrogen isotope discrimination, percent carbon, percent nitrogen, and carbon/nitrogen ratio. Leaves were oven‐dried at 65°C for 72 h and ground to homogenous powder using a Mixer Mill MM200 (Retsch, Haan, Germany) ball mill grinder, and analyzed at the Colorado Plateau Stable Isotope Laboratory at Northern Arizona University, Flagstaff, AZ, using a DELTA V Advantage isotope ratio mass spectrometer (Thermo Fisher Scientific, Waltham, MA), configured to a Finnigan ConFlo III, for automated continuous flow analysis of δ13C and %N using a Carlo‐Erba NC2100 elemental analyzer.

### 2.5 Environmental Data

To assess potential relationships between genetic, physiological parameters, and environmental conditions of seed sources, we obtained values for 19 bioclimatic variables and monthly solar radiation from WORLDCLIM at 1 km^2^ resolution for each seed source. Historical climate averages from 1970–2000 were used. Latitude, longitude, and elevation for each sample were also included in the environmental data matrix. The collection locations varied in environmental and geographical characteristics, including latitude, longitude, elevation, mean annual temperature, and mean annual precipitation (Table 1). Latitude, longitude, and elevation for each sample were also included for further analysis.

### 2.6 Statistical Analysis of Phenotypic Measurements

Each water‐use efficiency trait was tested for normal distribution and homoscedasticity using the Shapiro–Wilk test for normality and Levene's test of homogeneity of variance. Assimilation rate, SPAD, Thickness, d13C, C:N ratio were normally distributed and homoscedastic, whereas %N, SLA, WUE were transformed to a normal distribution using a log transformation. Traits such as: E, gs, Fresh Weight, d15N, %C were non‐normal and didn't conform to a normal distribution after transformations. For traits that conformed to parametric assumptions, a one‐way ANOVA with seed source as a random factor was conducted using the *aov* function in base R (R Core Team 2023) to determine differences among seed sources for each trait. Pairwise *t*‐tests using the holm correction were then used to determine which seed source differed between each other using the pairwise *t*‐test function found in the stats R library (R Core Team 2023). For traits that did not meet parametric assumptions, a Kruskal‐Wallis test with seed source as the factor, found within the stats library in R (R Core Team 2023), was used to determine differences among seed sources in each trait. Pairwise Wilcox tests with a holm correction were then used to determine where differences occurred between populations, using the pairwise Wilcox function in the stars library in R (R Core Team 2023). Each trait was then visualized using a box and whisker plot using functions within ggplot2 (Wickham 2016).

### 2.7 Correlations Between Environmental Data and Phenotypic Measurements

A correlation heatmap was used to examine the relationship among environmental variables. The environmental data was normalized by dividing each variable by its respective standard deviation, and a Euclidean distance matrix was calculated for the environmental data. A distance‐based linear model with a stepwise variable selection was then conducted, using functions found within Primer e (v7) (Clarke and Gorley 2015) by fitting the environmental data onto the physiological data to examine the amount of variation in physiological metrics that climate accounted for. The criterion for the distance‐based linear model was *R* ^2^. Longitude and Isothermality were determined to account for the most amount of variation as individual variables. Correlations between those two variables and each physiological trait were tested using Pearson's or Spearman's rank correlations. Correlations between elevation, mean annual precipitation, and physiological traits were also tested using the same methods.

### 2.8 Population Structure and Genetic Diversity

To mitigate the potential bias of linkage disequilibrium (LD) on population structure and admixture inferences, we pruned the dataset of 56,700 single nucleotide polymorphisms (SNPs) using the *Bcftools* v1.9 *prune* plugin (Danecek et al. 2011). LD pruning was performed by setting a minimum correlation threshold (*r* ^2^) of 0.2 within sliding windows of 1000 sites. This resulted in a reduced dataset of 17,563 SNPs, which was subsequently used for population clustering analyses. Principal Component Analysis (PCA) and Discriminant Analysis of Principal Components (DAPC) were carried out using the *Adegenet* package v2.1.10 (Jombart 2008). DAPC showed six PCs as the optimal number of PCs to retain. DAPC axis one accounted for 44.34% of variation and DAPC axis two accounted for 25.22% of variation. Classification accuracy of discriminant functions was 72.38%.

To further examine population structure, genomic admixture proportions were estimated using *Admixture* v1.3 (Alexander and Lange 2011) through the *AdmixPipe* v3.0 pipeline (Mussmann et al. 2020). We performed 20 independent runs for K values ranging from 1 to 10, applying a 20% cross‐validation (CV) procedure to assess model accuracy. The optimal number of genetic clusters (*K*) was determined using *EvalAdmix* (Garcia‐Erill and Albrechtsen 2020), and admixture plots were generated with *Clumpak* (Kopelman et al. 2015).

To evaluate isolation by distance (IBD), we performed a Mantel test using Nei's genetic distance (Nei 1972) calculated with the *vegan* v2.6–8, *dartR* v2.9.7, and *StAMPP* v1.6.3 packages (Dixon 2003; Gruber et al. 2018; Pembleton et al. 2013). Isolation by environment (IBE) was assessed using the *algatR* v1.0 package (Chambers et al. 2023), which employs an individual‐based approach leveraging environmental data from WORLDCLIM. Additionally, we computed Wright's pairwise *Fst* (Wright 1949; Cockerham 1984) between sampling localities using *StAMPP* to further explore population differentiation.

Genetic diversity was characterized based on heterozygosity levels from the full dataset of 56,700 SNPs, focusing exclusively on variant sites. Mean observed heterozygosity (*Ho*), expected heterozygosity (*He*), and the inbreeding coefficient (*Fis*) were estimated using the *dartR* and *StAMPP* packages. The number of private alleles per population was calculated with the *poppr* package v.2.9.6 in R (Kamvar et al. 2014), providing further insight into genetic variation across seed sources.

### 2.9 Genome‐Wide Association (GWAS) of Water Use Efficiency and Genome‐Environment Association (GEA)

For GWAS and GEA, the 56,700 SNPs dataset, without any LD pruning, was used for analysis. The rationale behind using the non‐LD pruned SNP dataset was to increase the chances of finding the causal genes or genes linked to those. A total of 96 individuals were both sequenced and phenotyped and were retained for the GWAS. Associations between SNP markers and water use efficiency traits were tested using the linear model (lm), univariate mixed linear model (lmm), and a multivariate linear mixed model (m‐lmm) from GEMMA (Zhou and Stephens 2012, 2014). Each physiological trait was tested individually first, and then a combination of traits including gas exchange, structural traits, and isotope traits was used to identify any other SNP markers that the univariate analysis did not identify. Due to the co‐linearity of some physiological traits, a subset of traits was chosen for the multivariate association test, which were Evapotranspiration, Specific Leaf Area, Chlorophyll Content, Leaf Thickness, and Carbon isotope discrimination. Population structure and kinship were accounted for in all GWAS analyses in GEMMA (Zhou and Stephens 2012, 2014). Bonferroni correction was used to correct for multiple testing with a corrected critical value of 8.818342e^−07^.

GEA analyses were conducted on 109 individuals as environmental data was collected for all sequenced individuals. To identify any potential outlier SNPs, PCadapt was used initially (Luu et al. 2017; Privé et al. 2020). PCadapt uses PCA based on Mahalanobis distance and Z‐scores of the contribution of each SNP to each principal component to detect SNPs that have a unique distribution relative to the majority of SNPs (Luu et al. 2017). The first 5 principal components were kept for analysis, and *p*‐values were converted to qvalues using the r package qvalue (Storey et al. 2023). *Q*‐values were then tested for significance using an alpha value of 0.05 with the Bonferroni correction method. GEA methods were used to cross‐validate one another. Candidate loci were thereby selected from coincident SNPs between PCadapt (Luu et al. 2017) and the mixed linear model from GEMMA (Zhou and Stephens 2012, 2014), and between PCadapt (Luu et al. 2017) and a redundancy analysis (RDA). Using different methods to cross‐validate GEA results and picking coincident SNPs as candidate loci helps to identify the SNPs that show the strongest relationship with environmental variables and thus are more robust associations. The RDA was conducted using 3 Bio‐climatic variables from WorldClim, Bioclimatic variable 3: Isothermality, Bioclimatic variable 6: mean temperature of the coldest month, and Bioclimatic variable 13: Precipitation of the wettest Month using the function found in the *vegan* R package (Oksanen et al. 2022). Climate variables for the RDA analysis were chosen based on variables identified in the distance‐based linear model conducted on physiological data and to avoid collinearity among variables in the RDA. For the mixed linear mixed model with GEMMA, each climate variable was tested individually as the response variable. All candidate loci were functionally annotated by obtaining 200 bp sequences around significant SNPs in both directions 5' and 3'. Functional annotation data was obtained by identifying orthologs found in the *Arabidopsis thaliana* TAIR10 genome using the blastx search function on ensemblplants.org (https://plants.ensembl.org/index.html).

## 3 Results

### 3.1 Population Structure, Gene Flow and Genetic Diversity

Population genomic analyses support one large genetic cluster with two seed sources that are differentiated from the rest of the island. PCA shows no distinct clustering among seed sources, most likely due to the low variation accounted for among the first two PCs (Figure S1). With 6 PCs used as optimal, DAPC shows that seed source QGUA is unique relative to the rest of the island and MAYG is somewhat unique (Figure 1). QGUA and MAYG are the two western seed sources, with QGUA being in the northwest portion of the island and MAYG being in the southwest (Figure 1). Interestingly, based on ordination distance, MAYG is much more similar to the eastern sources compared to its western neighbor. DAPC axes one and two account for over 70% of variation, indicating that the patterns observed in the DAPC (Figure 1) are more realistic of actual patterns on the landscape compared to the first two PCs (Figure S1). The presence of a large cluster represented by most of the seed sources and the clustering of MAYG (the southwestern most source) close to the eastern sources suggests that many seed sources have high gene flow across the island.

Admixture supported the potential for high gene flow across the island results with the best‐supported model indicating *K* = 1, suggesting extensive regional gene flow. Two clusters were the second‐best model (*K* = 2), and the number of Ks sequentially followed as the best fit. *K* = 2–4 is shown in Figure S2 to support the west‐to‐east differentiation seen in the DAPC (Figure S2). No significant isolation by distance (IBD) was detected, with an essentially null correlation coefficient (Figure S3). Similarly, isolation by environment (IBE) analysis revealed no significant relationship between climatic variability and genetic distance at the regional scale (Figure S3). Genetic differentiation, assessed using Nei's genetic distance and pairwise Fst, was very low, ranging from 0.0004 (BDUR‐LFRA) to 0.006 (PVIE‐MAYG), with an overall average of 0.0026. This supports the inference of extensive gene flow and near‐panmixia across the island (Tables S3 and S4). Genetic diversity was high and uniform across sites, with an average observed heterozygosity (Ho) of 0.77 and expected heterozygosity (He) of 0.48. The number of private alleles per site ranged from 774 at NAGU to 2006 at QGRA (Tables S3 and S4).

### 3.2 Physiological Analysis

**FIGURE 2:**
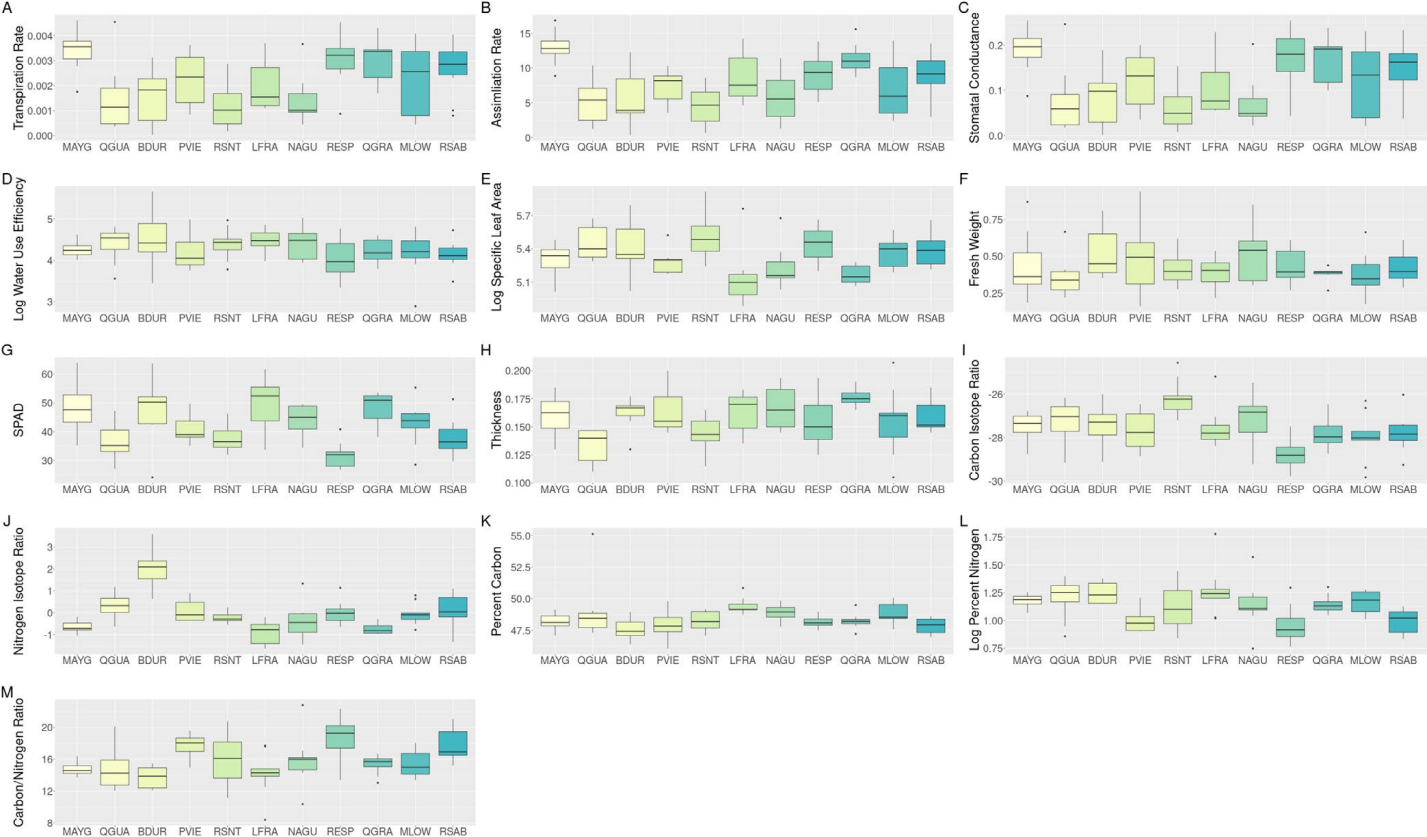
Box and whisker of water‐use efficiency traits where significant differences were found. Sites are ordered West to East with the westernmost seed source as the leftmost box and the easternmost is the rightmost box. All traits (A‐M) besides Log Water Use Efficiency and Fresh Weight showed significant differences with respect to seed source as a random factor. Specific test statistics can be found in Table S5.

Variation in gas exchange, structural traits and isotope traits suggests water use strategies may differ among seed sources. Scatterplots and boxplots show that MAYG generally had the highest gas exchange rates, which were similar to locations on the eastern side of the island (Figures 2 and S4). Interestingly, MAYG did not provide the higher values for all other traits, with individuals from that location being in the middle of the observed range for structural and isotope traits. QGRA also had some of the higher gas exchange rates and higher chlorophyll (SPAD) values; however, individuals from this location also had some of the lower SLA values, lower d15N values but mid‐range d13C values, indicating that while individuals from these two seed sources had higher gas exchange rates supported by the higher chlorophyll, they mitigated that potential water loss via structural traits and modulating stomatal opening (Figures 2 and S4). Other seed sources (e.g., PVIE) showed the exact opposite patterns. Individuals in the lower 50% of the gas exchange values supported by lower chlorophyll values were in the upper 50% of SLA and carbon isotope values, indicating that even though they had lower gas exchange rates, they kept their stomates open longer because of their low conductance and thus may lose more water. BDUR had some of the highest d15N values, suggesting higher photosynthetic capacity and more water use, but was in the 50% in SLA and the upper 50% of SPAD values (Figures 2 and S2). A large amount of within and among seed source variation was observed for most water‐use traits (Figures 2 and S4 Table S5). WUE and Fresh weight were the only traits measured without significant differences among seed sources. Strong trait–trait relationships were also observed with WUE, d13C and SLA being correlated with Percent Nitrogen (Figure S5). SPAD was significantly correlated with %N and Leaf thickness was significantly correlated with SLA (Figure S5). All statistical results are shown in Table S5.

**FIGURE 3:**
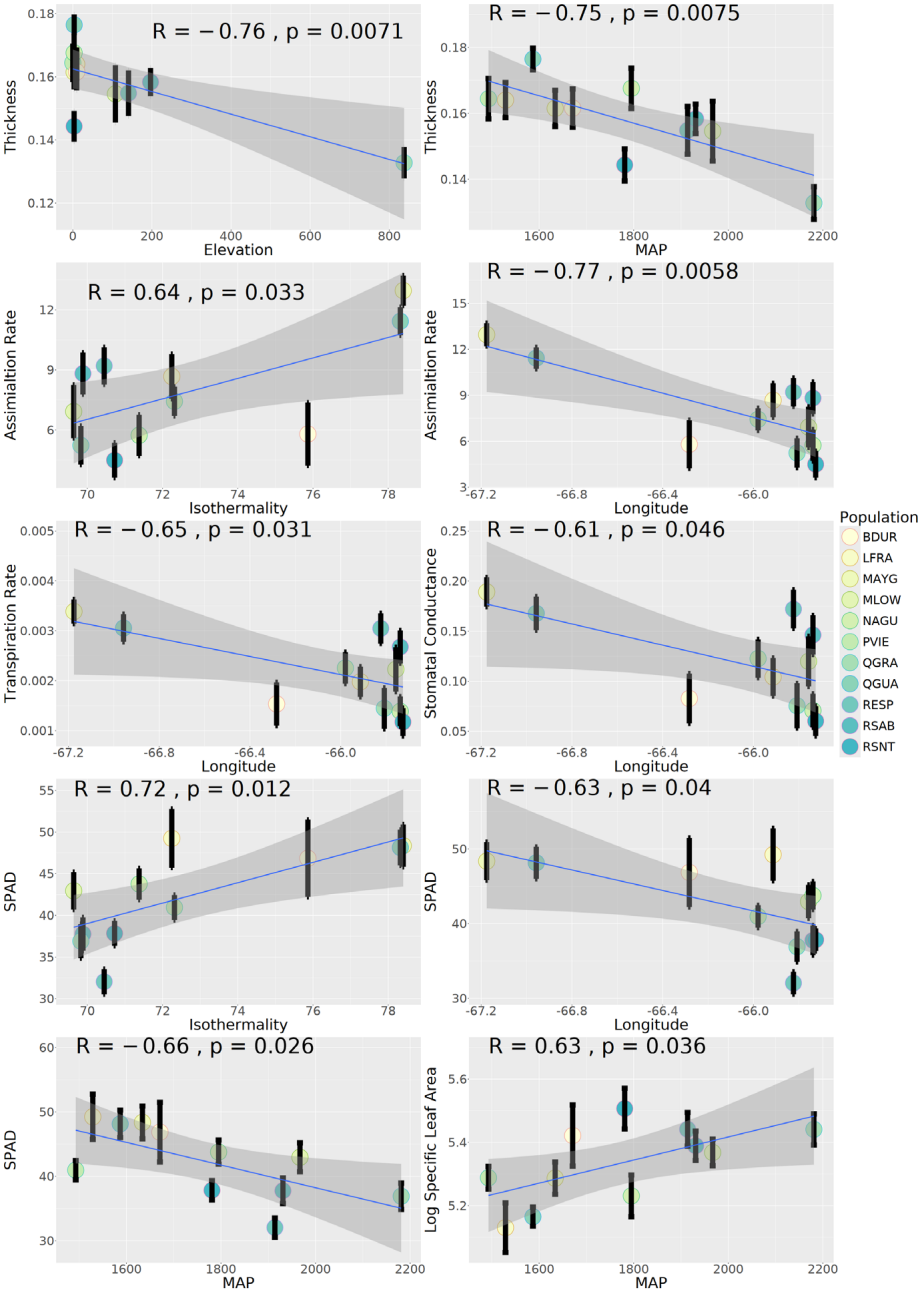
Correlation plots between water‐use efficiency traits and longitude, elevation and isothermality. Correlation (R) and *p*‐value are shown for each respective association. Blue lines represent a perfect linear relationship with grey areas as 95% confidence intervals.

Variation in water‐use efficiency traits among seed sources may be due to unique abiotic factors at each seed source, based on the results of the distance‐based linear model and individual correlations. The distance‐based linear model identified 10 variables associated with dissimilarity in water use. The best model produced by the distance‐based linear model had 10 variables (mean temperature of the wettest quarter, Isothermality, Solar radiation values for the months of March, April, June, July, August and October respectively as well as Latitude and Longitude) and an *R* ^2^ of 0.31292. Some individual traits were also significantly correlated with mean annual precipitation, Longitude, Elevation, and Isothermality; further supporting abiotic factors as potential drivers of variation in water‐use efficiency (Figure 3). Pairwise seed source tests (Table S6) suggested a slight longitudinal pattern for traits such as assimilation rate and leaf thickness as well, supporting some of these correlations. For many of the other traits such as SLA, Carbon Isotope Discrimination, Nitrogen Discrimination, %N, and %C, most of the significant pairwise differences were observed within the eastern half of the island, suggesting longitude and associated environmental factors might not influence every trait. SPAD, however, had both west–east differences and within the eastern half of the island, suggesting high variation in chlorophyll across the island.

The general patterns of differing water‐use efficiency strategies, the significant effect of geographic location on most traits, and the significant correlation of environmental variables suggest that individuals in these locations have varied strategies for water use by modulating gas exchange rates, nitrogen uptake, stomatal closures, and leaf structure. These overall patterns may be driven by a small number of genes, plasticity, and/or local variation in seed source climate (Figures 2, 3, and S4), rather than driven by strong genome‐wide differentiation (Figure 1). The fine scale pairwise differences within the eastern half of the island (Table S6) suggest that factors not measured in this study may also be influencing water‐use efficiency at a fine scale in the eastern half of Puerto Rico.

### 3.3 GWAS of Water Use Efficiency, GEA, and Outlier Analysis

**FIGURE 4:**
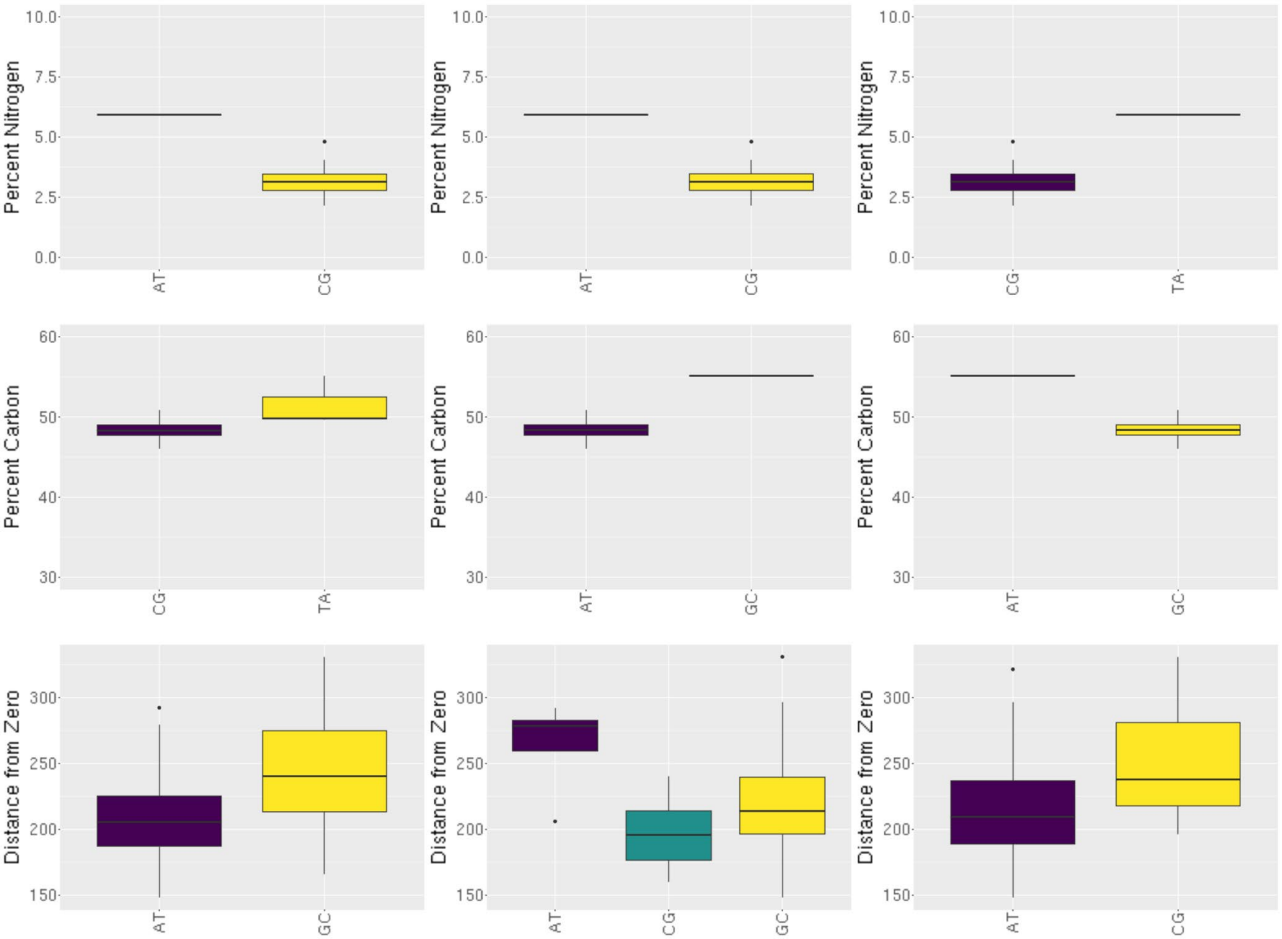
Genotypes of a subset of candidate loci SNPs identified from GEMMA. Observed genotypes are shown for each SNP. Raw values of percent carbon and percent nitrogen are presented. The distance from zero metric was calculated by taking the Euclidean distance for each trait from each individual relative to a zero matrix.

GEMMA identified 32 unique SNPs and phenotypic trait associations. From those, 14 SNPs were only associated with %N; one SNP was associated with both %C and %N; and 4 SNPs were only associated with %C. The multivariate linear mixed model identified 13 SNPs associated with the combination of transpiration rate, Specific Leaf Area, Chlorophyll content, Leaf Thickness, and Carbon isotope discrimination. There were no discrepancies of significant SNPs between the linear model and the univariate linear mixed model for any trait. Significant SNPs identified from GEMMA showed variation across genotypes for each trait (Figure 4), further supporting their association with each trait. The unique genotypes tended to have higher percent nitrogen and percent carbon. For the multi‐trait association, similar patterns were also shown with the unique genotypes tending to have a larger distance from the zero centroid (Figure 4). Fourteen out of thirty‐two candidate loci had *A. thaliana* orthologs and were associated with signal transduction, transcription regulation, transmembrane transport, and carbohydrate metabolism (Tables 2 and S7).

**TABLE 2:** Functional annotations of candidate loci identified by the GWAS and GEA.

TAIR 10 function	TAIR10 gene match	Number of SNPs	Variable/trait association
*Signal transduction*			
Zincin‐like metalloproteases family protein	AT1G67690	1	Precipitation of Wettest Quarter
Cysteine proteinases superfamily protein	AT3G02070	1	Mean Temp of the Coldest Month
Cysteine‐rich RECEPTOR‐like kinase	CRK8	8	%N, Precipitation of Wettest Quarter, E+SLA+SPAD+THICKNESS+D13C, Isothermality, Mean Temp of the Coldest Month, Precipitation of Wettest Quarter
Protein kinase superfamily protein	AT5G24080	1	Precipitation of Wettest Quarter
Lectin protein kinase family protein	AT1G34300	1	Isothermality
Serine/threonine‐protein phosphatase 7 long form‐like protein	AT1G48120	1	Isothermality
Leucine‐rich repeat protein kinase family protein	AT2G01210	1	Precipitation of Wettest Quarter
Leucine‐rich repeat (LRR) family protein	AT5G23400	1	Precipitation of Wettest Quarter
*Transcription regulation and translation*			
Class II aminoacyl‐tRNA and biotin synthetases superfamily protein	AT4G26870	1	%C
Basic helix–loop–helix (bHLH) DNA‐binding superfamily protein	AT1G68920	2	E+SLA+SPAD+THICKNESS+D13C
Myb/SANT‐like DNA‐binding domain protein	AT5G05800	1	Mean Temp of the Coldest Month
TLP8 description:tubby like protein 8	TLP8	1	Isothermality
Homeobox‐leucine zipper family protein/lipid‐binding START domain‐containing protein	PHV	1	E+SLA+SPAD+THICKNESS+D13C
ATP‐dependent RNA helicase	AT5G39840	1	Precipitation of Wettest Quarter
Zinc knuckle (CCHC‐type) family protein	AT1G75560	1	Mean Temp of the Coldest Month
Polynucleotidyl transferase, ribonuclease H‐like superfamily protein	AT3G01410	1	Precipitation of Wettest Quarter
Cleavage and polyadenylation specificity factor 73‐I	CPSF73‐I	1	Isothermality
BED zinc finger and hAT dimerization domain‐containing protein	DAYSLEEPER	1	%N
Villin 3	VLN 3	1	E+SLA+SPAD+THICKNESS+D13C
Ypt/Rab‐GAP domain of gyp1p superfamily protein	AT4G13730	1	Mean Temp of the Coldest Month
Forkhead‐associated (FHA) domain‐containing protein	AT1G75530	1	%N
*DNA and RNA methylation*			
Polynucleotidyl transferase, ribonuclease H‐like superfamily protein	AT5G42905	1	Isothermality
tRNA/rRNA methyltransferase (SpoU) family protein	AT4G17610	1	Mean Temp of the Coldest Month
DNAse I‐like superfamily protein	AT1G43760	1	Isothermality
*Transmembrane transport*			
Cyclic nucleotide gated channel 1	CNGC1	1	Precipitation of Wettest Quarter
Cyclic nucleotide gated channel 3	CNGC3	1	Precipitation of Wettest Quarter
Nucleobase‐ascorbate transporter 7	NAT7	1	%N
Transmembrane amino acid transporter family protein	AT1G48640	1	Isothermality
Xanthine/uracil permease family protein	AT5G62890	1	%N
HXXXD‐type acyl‐transferase family protein	AT5G17540	1	Mean Temp of the Coldest Month
HXXXD‐type acyl‐transferase family protein	AT5G23970	1	%N
*Primary and secondary metabolism*			
TTF‐type zinc finger protein with HAT dimerization domain‐containing protein	AT1G19260	1	Isothermality
2‐oxoglutarate (2OG) and Fe(II)‐dependent oxygenase superfamily protein	AT4G25300	1	Precipitation of Wettest Quarter
homogentisate phytyltransferase 1	HPT1	1	Isothermality
beta glucosidase 15	BGLU15	1	%C
*Repeat families*			
Ankyrin repeat family protein	AT5G04680	1	Mean Temp of the Coldest Month
Transducin/WD40 repeat‐like superfamily protein	AT5G19920	1	Mean Temp of the Coldest Month
Copia‐like polyprotein/retrotransposon	AT1G21280	3	Isothermality, Mean Temp of the Coldest Month
hAT transposon superfamily protein	AT4G08267	1	Precipitation of Wettest Quarter
*Uncharacterized proteins*			
Uncharacterized mito protein	ATMG00860	1	Isothermality
Uncharacterized mito protein	ATMG00810	1	Isothermality
protein_coding transcript_biotype	ATMG00850	1	Isothermality
protein_coding gene	AT2G22795	1	Mean Temp of the Coldest Month
protein_coding gene	AT1G37113	1	Mean Temp of the Coldest Month
protein_coding gene	KTI5	1	Mean Temp of the Coldest Month

*Note:* The leftmost column indicates the function of the *A. thaliana* ortholog identified from the candidate loci, followed by the gene name of the identified ortholog in *A. thaliana*. The GWAS/GEA column indicates whether the ortholog was found from either the respective GWAS or GEA analyses, or both. The number of SNPs column shows the total number of unique SNPs that matched each respective ortholog, either from the GWAS and the GEA combined. The Variable/Trait Association column indicates which phenotypic trait(s) and climate variables the candidate loci were associated with. E‐values can be found in Table S7.

PCadapt identified 11,425 outlier SNPs prior to correction and 6617 post Bonferroni correction for multiple testing. For the GEA analyses, the RDA identified 588 candidate loci, 232 SNPs that were most strongly associated with Isothermality, 210 SNPs that were most strongly associated with the mean temperature of the coldest month, and 147 that were most strongly associated with precipitation of the wettest quarter. The mixed linear model in GEMMA identified 4 candidate SNPs, each of whom was associated with a different environmental variable and combinations of those (precipitation of the driest quarter and annual precipitation, temperature annual range, mean diurnal range and temperature annual range, max temperature of the warmest month). There were 2 SNPs that were coincident among the GEMMA models and PCadapt, and 185 SNPs coincident among the RDA and Pcadapt. From these 185, only 45 had orthologs found in the TAIR 10 *A. thaliana* genome, and were associated with signal transduction, transcription regulation, DNA and RNA methylation, transmembrane transport, and primary and secondary metabolism (Tables 2 and S7).

### 3.4 Variation in Environment and Genome × Environment Space

**FIGURE 5:**
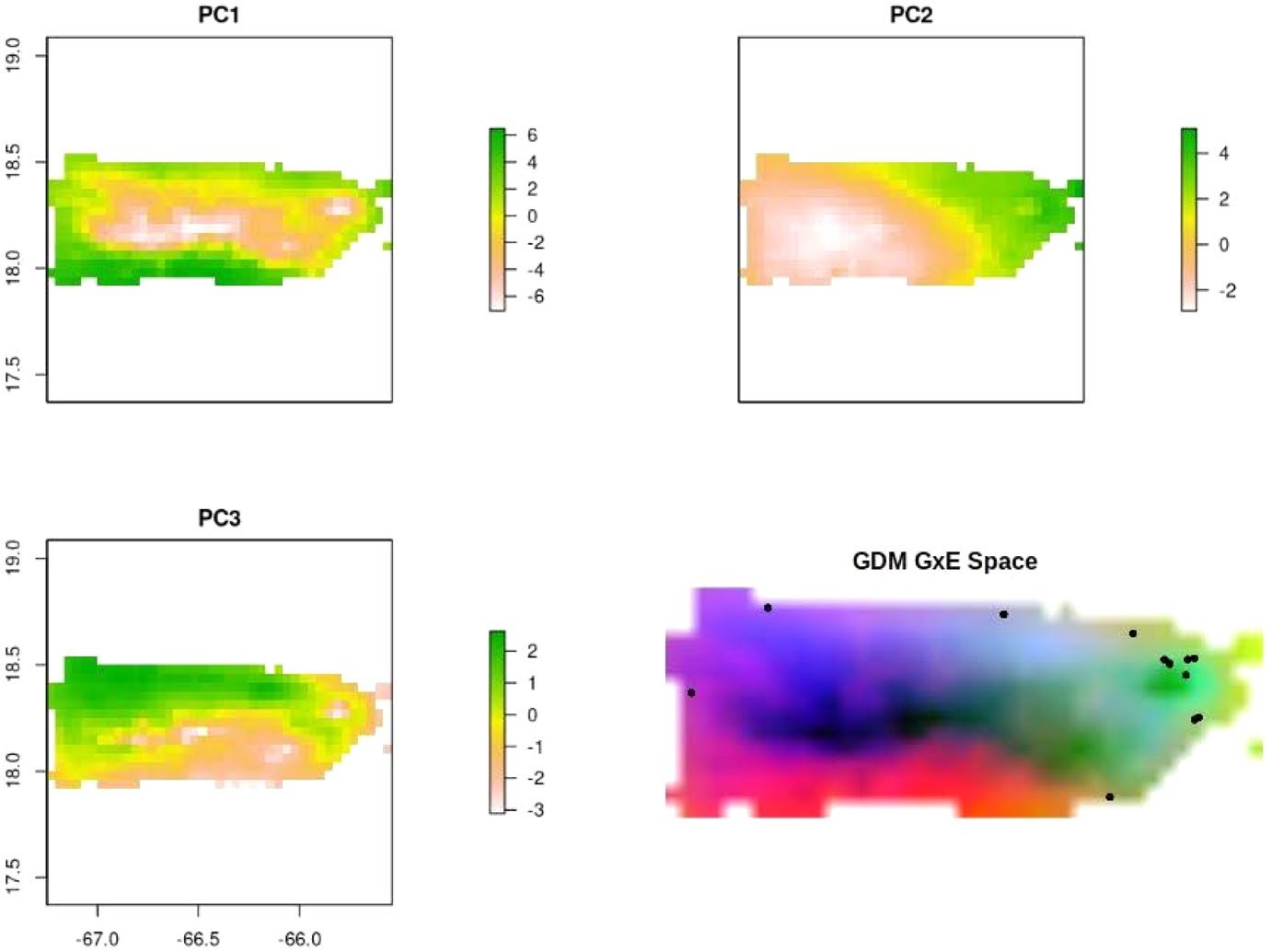
Environmental PCA similarities from WorldClim bioclimatic variables across the island. The more similar the color is, the more similar the eigenvectors of each PC are, suggesting a more similar environmental makeup. Generalized Dissimilarity Model projections of GxE space across the island. Black dots are collection locations used to train the model. Pixel color represents GxE space, the more similar a pixel color is, the more similar the projected GxE is estimated to be.

The PCA of environmental variables across the island shows that differences in temperature and annual precipitation have strong clines along the coastal to interior portions of the island, based on the projection of PC1 and the variable loadings of PC1 (Figure 5 and Table S8). Strong clines are observed in the northeastern portion of the island, where most sampling locations occur (Figure 5 and Table S8). PC2 shows a stark west to east cline in precipitation seasonality and temperature variables associated with temperature range or of wettest and driest quarters (Figure 5 and Table S8). PC3 also shows a west to east cline of similar variables of PC2, with some more heterogeneity in the northeastern portion of the island (Figure 5 and Table S8). The generalized dissimilarity model (GDM) also shows strong west to east variation in projected genome × environment space (Figure 5). Based on the lack of strong genetic differentiation observed in this study (Figure 1, Tables S3, S4), the main contributor of variation in the projected GxE space is most likely the strong environmental variation observed within the island.

## 4 Discussion

### 4.1 Role of Genetics and the Environment in the Maintenance of Trait Variation in Natural Populations of Puerto Rican *P. officinalis*

Overall, our results suggest a major role of local environmental pressures in maintaining phenotypic trait variation in the species and the potential for local adaptation in a small number of genes despite the presence of high gene flow among populations. We observed generally weak population structure, low pairwise Fst values, high genetic diversity, and the absence of isolation by distance and isolation by environment; all of which suggest a near panmictic population structure with extensive levels of gene flow and very little differentiation in Puerto Rican *P. officinalis* populations. Historically, high gene flow has been believed to counteract the effects of local adaptation, leading to minimal phenotypic variation across geographic areas (Balkau and Feldman 1973; Blanquart and Gandon 2011; Haldane 1930; Lenormand 2002). The lack of strong genetic differentiation across most of the island observed in our study would suggest high levels of gene flow among populations; therefore, genome‐wide local adaptation is potentially mitigated (Balkau and Feldman 1973, Blanquart and Gandon 2011, Haldane 1930, Lenormand 2002). However, our results suggest the potential for a small number of genes to be involved in local adaptation based on trait differences among genotypes (Figure 4), significant SNPs associated in the GEA, and candidate loci associated with key biological processes in relation to growth, development, and biotic and abiotic stress (Table 2). Recent studies in plants and animals have shown evidence of local adaptation even with high gene flow that has led to variation in phenotypes (Comeault et al. 2015; Fitzpatrick et al. 2015; Gonzalo‐Turpin and Hazard 2009; Joron et al. 2011; Laurent et al. 2016; Muir et al. 2014; Sambatti and Rice 2006) potentially due to strong natural selection maintaining genetic variation specific to each local environment (Brown et al. 2007; Kittelson and Maron 2001; McKay et al. 2001; Sambatti and Rice 2006; Vekemans and Lefèbvre 1997).

The relationship between local environment, genes, and phenotypes observed in our study supports that the local environment is a strong selection pressure maintaining variation in a small number of genes. We observed differences in the projected GxE space (Figure 5), in environmental principal components (Figure 5 and Table S8) and direct environmental metrics (Tables 1 and S2). Furthermore, we observed correlations between leaf traits and mean annual precipitation, longitude, elevation, and isothermality (Figure 3), which suggest strong environmental selection pressures acting on leaf trait variation in Puerto Rico. Environmental clines shown in the environmental PCA (Figure 5 and Table S8) suggest strong environmental variation among closely located seed sources. Further evidence is needed to see if there is a congruent phenotypic response in natural populations in comparison to our common garden study. Environmental and geographical factors influencing selection on foliar traits, and the importance of plasticity shaping other phenotypic traits could be better understood with replicated field common gardens across the longitudinal gradient or reciprocal transplant experiments.

### 4.2 Functional Annotation of Candidate Loci

The results of our GWAS and GEA analyses have identified a group of 59 candidate genes involved in key biological processes such as signal transduction, transcription regulation, DNA and RNA methylation, transmembrane transport, primary and secondary metabolism, and transposon activity. These candidate genes have never been previously reported in the species. From the genes involved in signal transduction, one gene was associated with multiple traits and environmental variables. Cysteine‐rich RECEPTOR‐like kinase (CRK8) was found to be associated with %N, E, SLA, SPAD, Thickness, D13C, Isothermality, mean temperature of the Coldest month, and Precipitation of the wettest quarter in *P. officinalis* . CRK8 is involved in amino acid phosphorylation and has been transcriptionally induced in response to abiotic stress conditions such as drought, salicylic acid, ozone, UV light, and salt treatments, as well as pathogen infection in several plant species (Bourdais et al. 2015; Yeh et al. 2015; Quezada et al. 2019). Similarly, Leucine‐repeat (LRR) genes (e.g., AT2G01210 and AT5G23400 in this study) are involved in various stress responses including drought and salt stress in species such as peanut (Wang et al. 2024) and rice (Li et al. 2024).

Another important group of candidate genes with key biological processes is the one involved in transcription regulation. The Basic‐helix–loop–helix (bhLH) DNA‐binding protein (AT1G68920) was associated with E, SLA, SPAD, Thickness, and Carbon Isotope Discrimination in *P. officinalis*. Genes from this family are involved in many physiological processes, including growth, development, and stress response in Arabidopsis (Hao et al. 2021). Villin 3 (VLN3) and Homeobox‐leucine zipper protein (PHV) were also associated with E, SLA, SPAD, Thickness, and Carbon Isotope Discrimination in *P. officinalis*. VLN3 has been linked to stomatal immunity (closing stomatal to restrict pathogen entry) in Arabidopsis (Zou et al. 2021), and stress and developmental processes in soybean (Zhou et al. 2023).

### 4.3 Effect of Seed Source Environment on Photosynthetic Rate and Water‐Use Efficiency

Our results highlight the importance of seed source on foliar trait variation and provide evidence of strong environmental selection pressures in the species. More specifically, precipitation and temperature of seed sources strongly predict photosynthetic rate and water use efficiency in *P. officinalis* , as seen in other forest tree studies (Fyllas et al. 2009; Wright et al. 2005a). In our study, gas exchange rate and SPAD were positively correlated with isothermality, indicating higher photosynthetic rates with a larger diurnal temperature range. Isothermality is also strongly positively correlated with mean annual temperature (MAT) for our study area, so this increase in photosynthetic rate could be due to overall warmer temperatures in areas with higher isothermality (Figure S6). Similar relationships between photosynthetic rate and temperature have been seen throughout the literature (Asner et al. 2017; Fyllas et al. 2009; Heilmeier 2019; Wright et al. 2005a). Increases in mean annual precipitation (MAP) were associated with increases in SLA as well as decreases in SPAD and Thickness. More precipitation would lead to a decreased need for high water‐use efficiency, which explains the higher SLA and the lower leaf thickness, as individuals with abundant water resources would need to invest less energy in limiting foliar water loss (Asner et al. 2017; Heilmeier 2019). Similar relationships between the inverse of SLA (leaf mass area) and MAP have been shown in the literature as well (Fyllas et al. 2009; Wright et al. 2005b; Asner et al. 2017). One would think that with more water, individuals would be more photosynthetically active, but lower photosynthetic capacity with higher MAP has been seen in the literature before (Santiago et al. 2004). The decrease in SPAD with increasing MAP could be due to the strong negative correlation between MAP and MAT in our study area, leading to areas with higher rainfall having generally cooler temperatures (Figure S6), which would lead to a generally lower photosynthetic capacity and rate (Asner et al. 2017; Fyllas et al. 2009; Heilmeier 2019; Wright et al. 2005a). Photosynthetic rate and water use efficiency are two extremely important processes for plant growth and survival. The relationships between climate and photosynthetic rate and water‐use efficiency have been documented in field studies (Asner et al. 2017, Fyllas et al. 2009, Heilmeier 2019, Wright et al. 2005a). Our study in a well‐watered common garden supports many of those established relationships on the effect of seed source environment on phenotypic trait variation in *P. officinalis* . Future studies should explore the roles of phenotypic plasticity and maternal effects on Puerto Rican *P. officinalis* and compare them with previous studies of trait variation in a variety of plant species (Galloway 2005; Galloway and Etterson 2007; Latzel et al. 2014; Sultan et al. 2009). Regardless of the mechanisms behind these processes, the trait‐environment relationships further highlight the importance of choosing seed sources for restoration efforts, as the collection environment of the seed source seems to play an extremely important role in photosynthetic and water‐use trait variation.

### 4.4 Trade‐Off Between Water Use Efficiency and Photosynthetic Capacity and Other Foliar Trait Relationships

Foliar traits were not only related to environmental and geographic characteristics, but significant relationships among traits were observed, many of which are common in other mangroves and tropical trees. Based on trait correlations, we observed a general trade‐off between water use efficiency and photosynthetic capacity. Significant relationships among SLA, leaf thickness, and SPAD were observed, which supports results found in Marenco et al. (2009), Jifon et al. (2005), and Yamamoto et al. (2002). SLA was negatively correlated with assimilation rate and SPAD; similar relationships have been found in other tropical tree species (Marenco et al. 2009; Santiago and Wright 2007) and crops (Nageswara Rao et al. 2001; Nigam and Aruna 2008). SLA was positively correlated with foliar percent nitrogen, which has also been seen in other mangroves (Quadros et al. 2021). We also observed positive relationships between assimilation rate and transpiration rate, seen in many other mangrove species (Maruyama et al. 1997). Other metrics of water use efficiency (Instantaneous WUE and d13C) were significantly and positively correlated with metrics of photosynthetic capacity (%N), which has been seen in other legumes (Adams et al. 2016), further supporting the general trade‐off between WUE and photosynthetic rate. Interestingly, Instantaneous WUE and d13C had no significant correlation with each other, which has been seen in other plant studies (Chaves et al. 2007; de Souza et al. 2005; Poni et al. 2009). Bchir et al. (2016) found that the relationship between d13C and WUE varied year to year, suggesting the relationship between d13C and WUE determined from gas exchange is dependent upon factors associated with each individual growing season.

### 4.5 Considerations for Future Water Use Efficiency Assessment in Puerto Rican *P. officinalis*

In terms of the assessment of seed sources for future restoration efforts, our study helps to show which traits can provide a quality assessment of water‐use efficiency for minimal cost and effort. Based on significant trait correlations shown in Figure S5, trait correlations can help identify the most cost‐effective approach. As mentioned above, there was a non‐significant correlation between instantaneous water‐use efficiency and carbon isotope ratio, indicating that the snapshot measurements provided by the portable gas‐exchange measurement system were not fully representative of the growing season, which has been found in a variety of species (Dixit et al. 2022). This would suggest that prioritizing stable isotope measurements over gas exchange measurements may be more worthwhile in terms of holistic phenotype assessment. Chlorophyll content (SPAD) was significantly correlated with %N, suggesting it could be an effective analog for photosynthetic capacity in Puerto Rican *P. officinalis* . SPAD monitors are available at a lower cost compared to the cost required for stable isotope analysis. Previous studies in mangrove species have also suggested chlorophyll content is an effective proxy for photosynthetic capacity (Neres et al. 2020) and for plant and ecosystem functioning (Heenkenda et al. 2015; Pastor‐Guzman et al. 2015). Leaf thickness was correlated with SLA, suggesting it is a viable alternative for assessing water‐use efficiency. Relationships between leaf thickness and SLA were also seen in other tropical tree species (Marenco et al. 2009). Measuring leaf thickness is much less time‐consuming than measuring SLA and does not require destructive sampling of a leaf. Previous literature in other species had also shown the relationship among leaf thickness and other water‐use efficient traits (Cao et al. 2020), supporting the effectiveness of leaf thickness as an easier and cheaper analog for water‐use efficiency. Based on the results of our study, the most parsimonious and cheapest phenotypic assessment for water‐use efficiency could just entail SPAD and leaf thickness measurements. If funds are available, adding stable isotope analysis can add valuable information as well.

### 4.6 Implications for Restoration

While our study was conducted in well‐watered conditions, based on the results of our foliar traits, we were able to identify a starting point for climate‐smart seed sourcing guidelines for Puerto Rican *P. officinalis* . We identified 3 seed source locations that would be useful for restoration areas with abundant water (MAYG, BDUR and RSNT), based on their high gas exchange rates, higher %N, and more negative carbon isotope ratio. The phenotypic profile of these three seed sources would suggest that they are less water‐use efficient but can be faster growing. For restoration areas where drought is expected, LFRA and RESP would be good seed source locations as they have higher water‐use efficiency based on SLA and carbon isotope ratio as well as lower photosynthetic capacity based on %N, thereby more likely to be slower growing. It would appear keeping east–west seed sources specific to their respective longitude would keep similar genetic makeup on the landscape relative to their current local environment, which could be useful for the short term. However, with projected changing climates, the results of GEAs help to provide specific alleles that are associated with specific climate regimes and so seed sources can also be chosen based on their association to new climates. Western and eastern areas could serve as genetic reserves for the island and allow for new alleles to be exchanged when planted in new areas. Being that genetic differentiation didn't necessarily lead to equal phenotypic differentiation in QGUA, this suggests QGUA could serve as a reserve of unique alleles that don't drastically alter phenotypes. Increasing genetic diversity without drastically altering phenotypes could help build a reserve of heterozygosity that the species could use to adapt to future stressors faster than they normally would. While our study does provide a broad‐spectrum assessment of water use phenotypes, future studies should still examine the performance of these collection sources in field conditions to better understand their effectiveness in restoration applications. Future studies should also examine how offspring from assisted gene flow perform in field settings to see if increasing heterozygosity via assisted gene flow creates more robust offspring.

## Disclosure

PERMISSION TO REPRODUCE MATERIAL FROM OTHER SOURCES: All material presented is from our study alone, unless otherwise cited.

## Ethics Statement

The authors have nothing to report.

## Consent

The authors have nothing to report.

## Conflicts of Interest

The authors declare no conflicts of interest.

## Supporting information


**Figure S1.** PC1 and PC2 of LD filtered SNP dataset. PC1 accounts for 1.5% of variation. PC2 accounts for 1.4% of variation.

**Figure S2.** Admixture proportions for *K* = 2–4, separated by east and west populations. Although *K* = 1 was the best *K* for our dataset, *K* = 2–4 are presented to help visualize that there is some slight genetic variation between the east and west seed sources of the island.

**Figure S3.** Isolation by distance and environment. Isolation by distance (top left panel). Isolation by environment along PC 1 (top right panel), by environmental PC 2 and 3 (bottom panels). Estimated correlations are shown in blue lines.

**Figure S4.** Scatterplots of paired water use traits. Gas exchange A‐B, Structural traits, C‐D, Isotope Traits E‐F. Ellipsoid represents the 95% Confidence Region.

**Figure S5.** Heatmap of correlation between water‐use efficiency traits. Blank boxes indicate a non‐significant correlation. All correlations were tested using spearman rank correlation.

**Figure S6.** Heatmap of correlations between Lat, Long, Elevation and Bioclimatic variables. Blank boxes indicate non‐significant correlations.

**Table S1.** Basic Sequence quality information from fastp output. Sample column shows sample name. Total reads before shows initial amount of raw reads received. Percent in Q20 shows the percent of nucleotides across all reads in the Q20 or higher. Retained Reads post filtering shows the number of reads kept after all filtering using a Q30 or high criteria, with the percentage of reads in kept following Q30 filtering in the final column.
**Table S2**. Locations of seed sources used in our study. In‐text shorthand name used for each seed source are shown in Collection site code column. Lat, Long and all World Clim Bioclimatic and Solar radiation values. environmental data downloaded from WorldClim at a 1^2km resolution. wc2_1_30s_bio_1 = Annual Mean Temperature. wc2_1_30s_bio_2 = Mean Diurnal Range (Mean of monthly (max temp—min temp)); wc2_1_30s_bio_3 = Isothermality (BIO2/BIO7) (×100); wc2_1_30s_bio_4 = Temperature Seasonality (standard deviation ×100); wc2_1_30s_bio_5 = Max Temperature of Warmest Month; wc2_1_30s_bio_6 = Min Temperature of Coldest Month; wc2_1_30s_bio_7 = Temperature Annual Range (BIO5‐BIO6); wc2_1_30s_bio_8 = Mean Temperature of Wettest Quarter; wc2_1_30s_bio_9 = Mean Temperature of Driest Quarter; wc2_1_30s_bio_10 = Mean Temperature of Warmest Quarter; wc2_1_30s_bio_11 = Mean Temperature of Coldest Quarter; wc2_1_30s_bio_12 = Annual Precipitation; wc2_1_30s_bio_13 = Precipitation of Wettest Month; wc2_1_30s_bio_14 = Precipitation of Driest Month; wc2_1_30s_bio_15 = Precipitation Seasonality (Coefficient of Variation); wc2_1_30s_bio_16 = Precipitation of Wettest Quarter; wc2_1_30s_bio_17 = Precipitation of Driest Quarter; wc2_1_30s_bio_18 = Precipitation of Warmest Quarter; wc2_1_30s_bio_19 = Precipitation of Coldest Quarter wc2_1_30s_srad_1—wc2_1_30s_srad_012 Represent average monthly solar radiation values for January (1) to December (12) respectively.
**Table S3**. Dragonsblood tree ( *Pterocarpus officinalis* ) pairwise Fst differentiation values across twelve sampling sites in Puerto Rico. Site code is from the shorthand population code.
**Table S4**. Dragonsblood tree ( *Pterocarpus officinalis* ) genetic diversity estimates obtained at twelve sampling sites in Puerto Rico. Site code is from the shorthand population code. The 17,056 SNP dataset was used to assess population structure and the 56,700 dataset was used for the GWAS and GEA analyses. Heterozygosity metrics are shown with the mean plus or minus the standard deviation. Ho represents the observed heterozygosity. uHe represented the unbiased expected heterozygosity. PA represents the private alleles found and Fis represents the inbreeding coefficient.
**Table S5**. Test statistic for each water‐use efficiency traits. F statistics are from the one‐way ANOVA, *χ* ^2^ statistics are from the chi‐square test. Long Cor column shows significant correlations between traits and longitude. Elev Cor column shows significant correlations between elevation and traits. Iso Cor Column shows significant correlations between traits and isothermality. Columns LFRA to PVIE show the group means and standard error of the mean for each trait respective to each seed source.
**Table S6**. Pairwise Comparisons for each water‐use trait. Traits are separated in tables A‐K with the trait listed in the leftmost column in bold. For traits that conformed to parametric assumptions (Assimilation Rate, Log Specific LeafArea, SPAD, Thickness, d13C, %N and C/N Ratio) a pairwise *t*‐test with holm correction was used. For Traits that did not meet parametric assumptions (Transpiration, stomatal conductance, d15N, %C), pairwise wilcox tests with holm correction was used. Shorthand location names are in the 1st row and 1st column of each table indicating the specific pairwise comparison.
**Table S7**. Full BlastX Results with Arabidopsis orthologs for candidate genes identified from GWAS and GEA. Leftmost column indicates the sequence coordinates from the *P. macrocarpa* genome used for functional annotation; followed by the function of the *A. thaliana* ortholog identified from the candidate SNP; followed by the gene ID of the identified ortholog in *A. thaliana*. E‐val, Length, score and orientation are all metrics supplied by the BlastX algorithm. The Variable association column indicates whether the ortholog was found from either the respective the GWAS or GEA analyses. Function column provides information on functional annotation of each gene.
**Table S8**. Worldclim bioclimatic variable contributions to the three principal component axes. Information regarding each bioclimatic variable can be found at https://www.worldclim.org/data/bioclim.html. PC loadings for each variable are presented to help show the main drivers of environmental variation across the island of Puerto Rico.

## Data Availability

Whole‐genome sequences: NCBI Bioproject accession PRJNA1168980.
