# Supplementary figures and images for: Genomic and Physiological Basis of Structural and Foliar Trait Variation in Tropical Species Pterocarpus officinalis : Implications for Restoration in Future Drier Climates
Source: Evol Appl. 2025 Apr 28;18(5):e70102. doi: 10.1111/eva.70102 (PMC12037992; doi:10.1111/eva.70102)

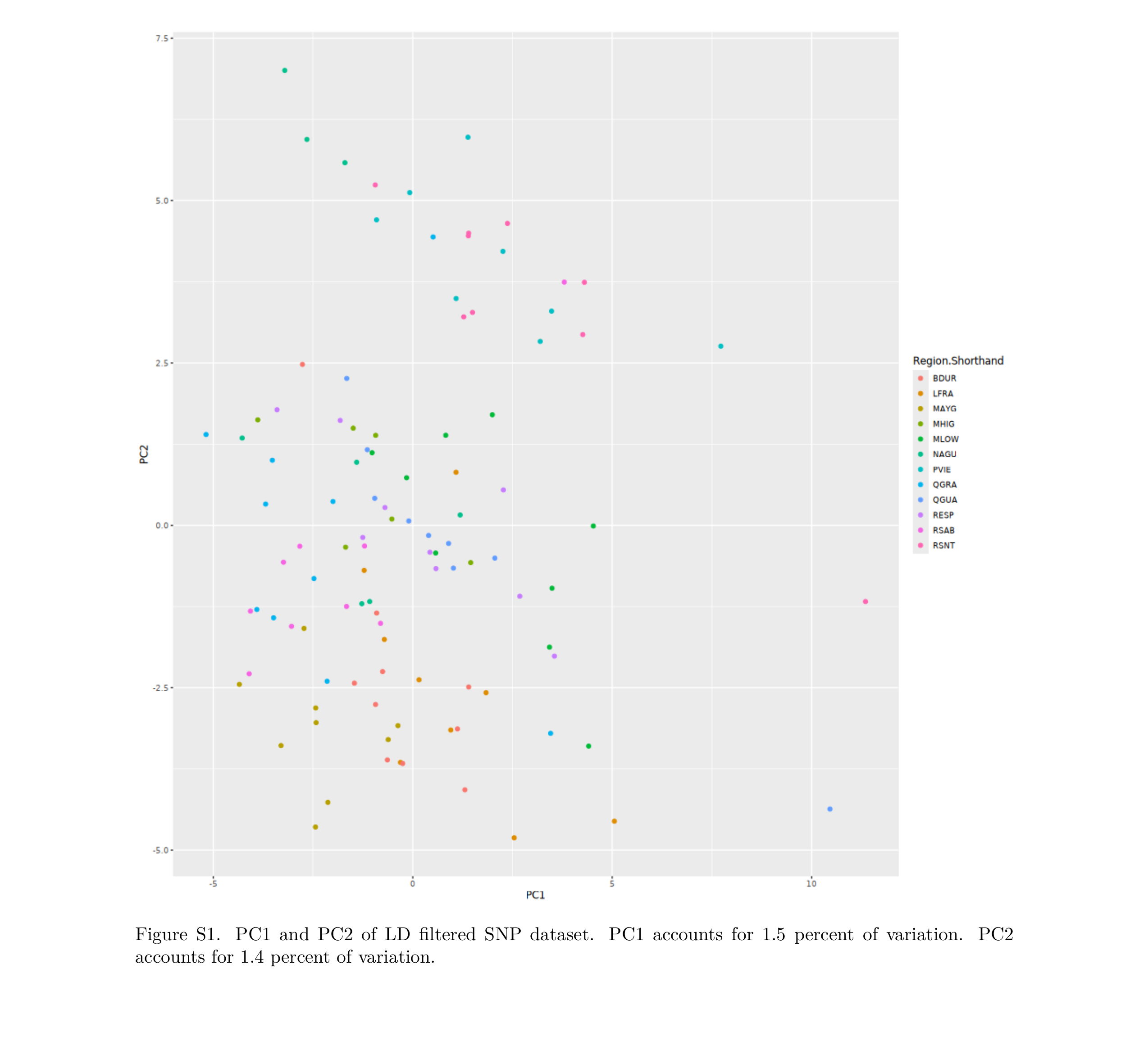

Supplement: Supplementary file 1 — Figure S1. PC1 and PC2 of LD filtered SNP dataset. PC1 accounts for 1.5% of variation. PC2 accounts for 1.4% of variation. [file EVA-18-e70102-s004.png]

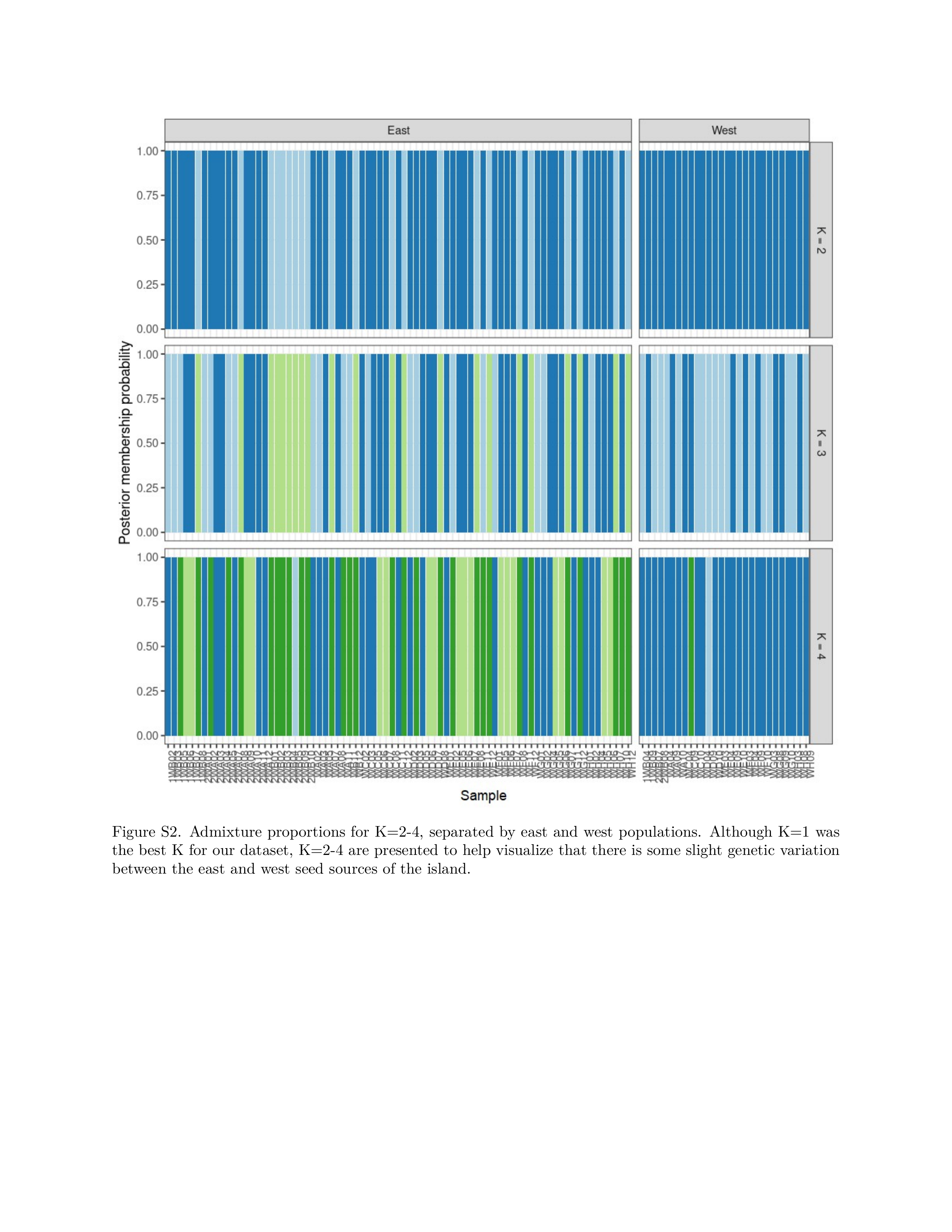

Supplement: Supplementary file 2 — Figure S2. Admixture proportions for K = 2–4, separated by east and west populations. Although K = 1 was the best K for our dataset, K = 2–4 are presented to help visualize that there is some slight genetic variation between the east and west seed sources of the island. [file EVA-18-e70102-s005.png]

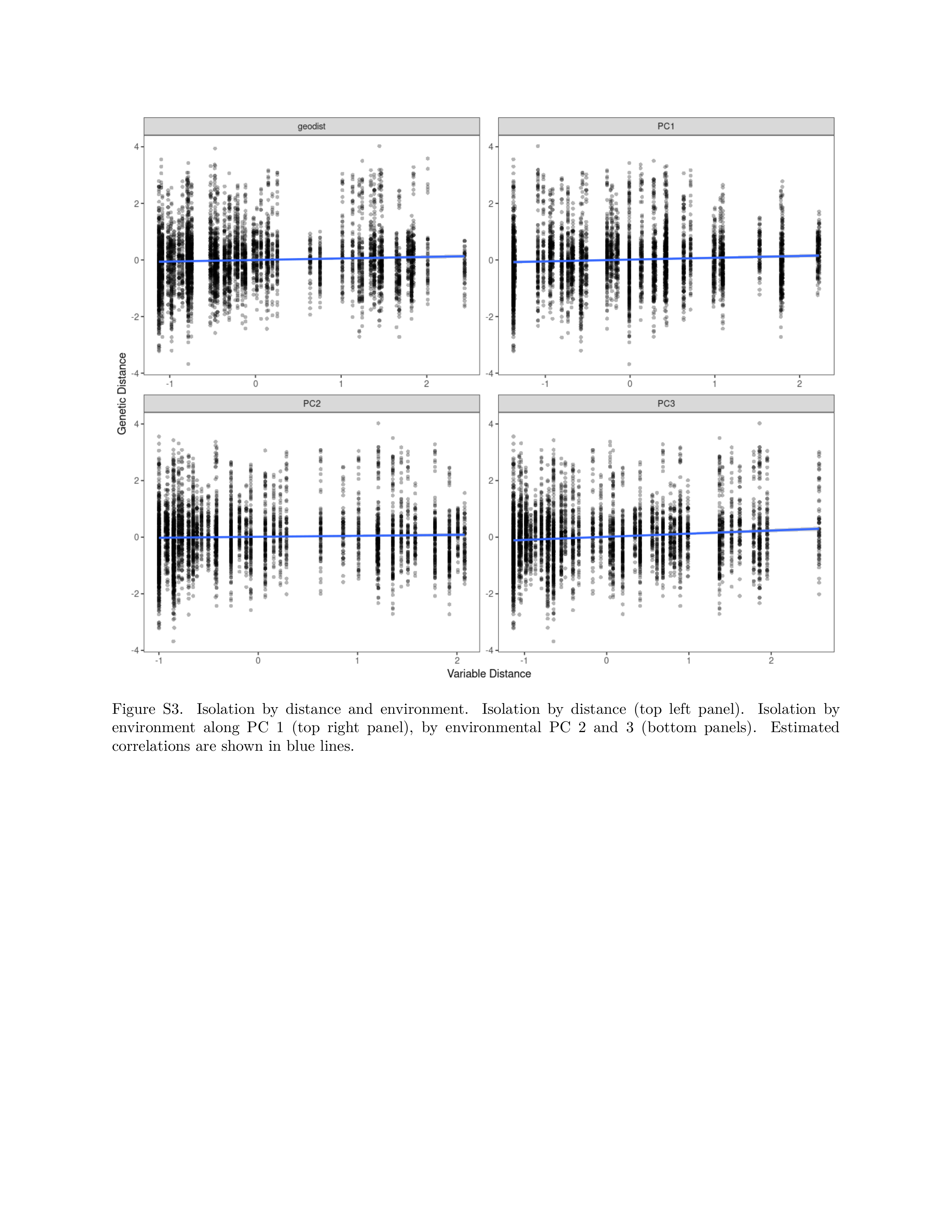

Supplement: Supplementary file 3 — Figure S3. Isolation by distance and environment. Isolation by distance (top left panel). Isolation by environment along PC 1 (top right panel), by environmental PC 2 and 3 (bottom panels). Estimated correlations are shown in blue lines. [file EVA-18-e70102-s002.png]

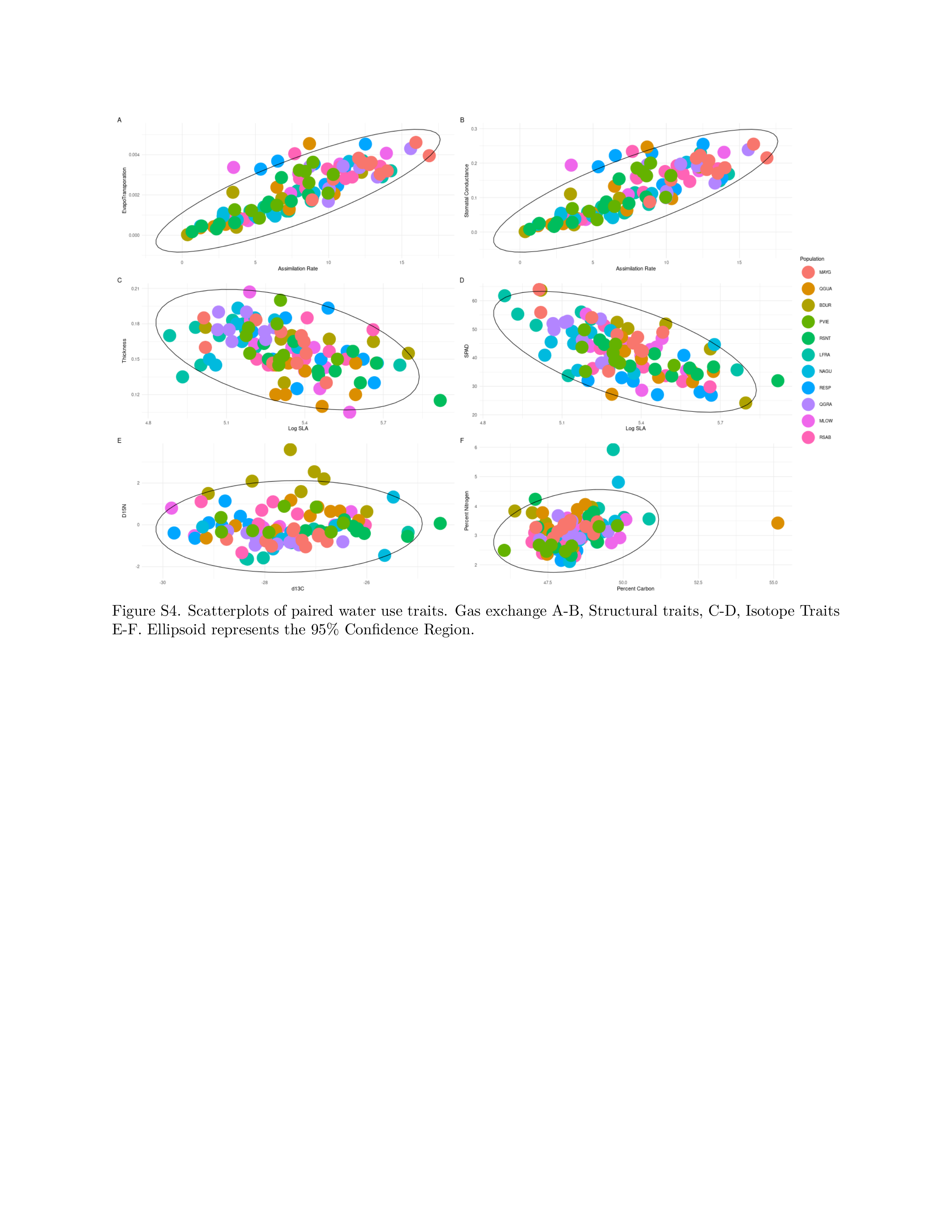

Supplement: Supplementary file 4 — Figure S4. Scatterplots of paired water use traits. Gas exchange A‐B, Structural traits, C‐D, Isotope Traits E‐F. Ellipsoid represents the 95% Confidence Region. [file EVA-18-e70102-s003.png]

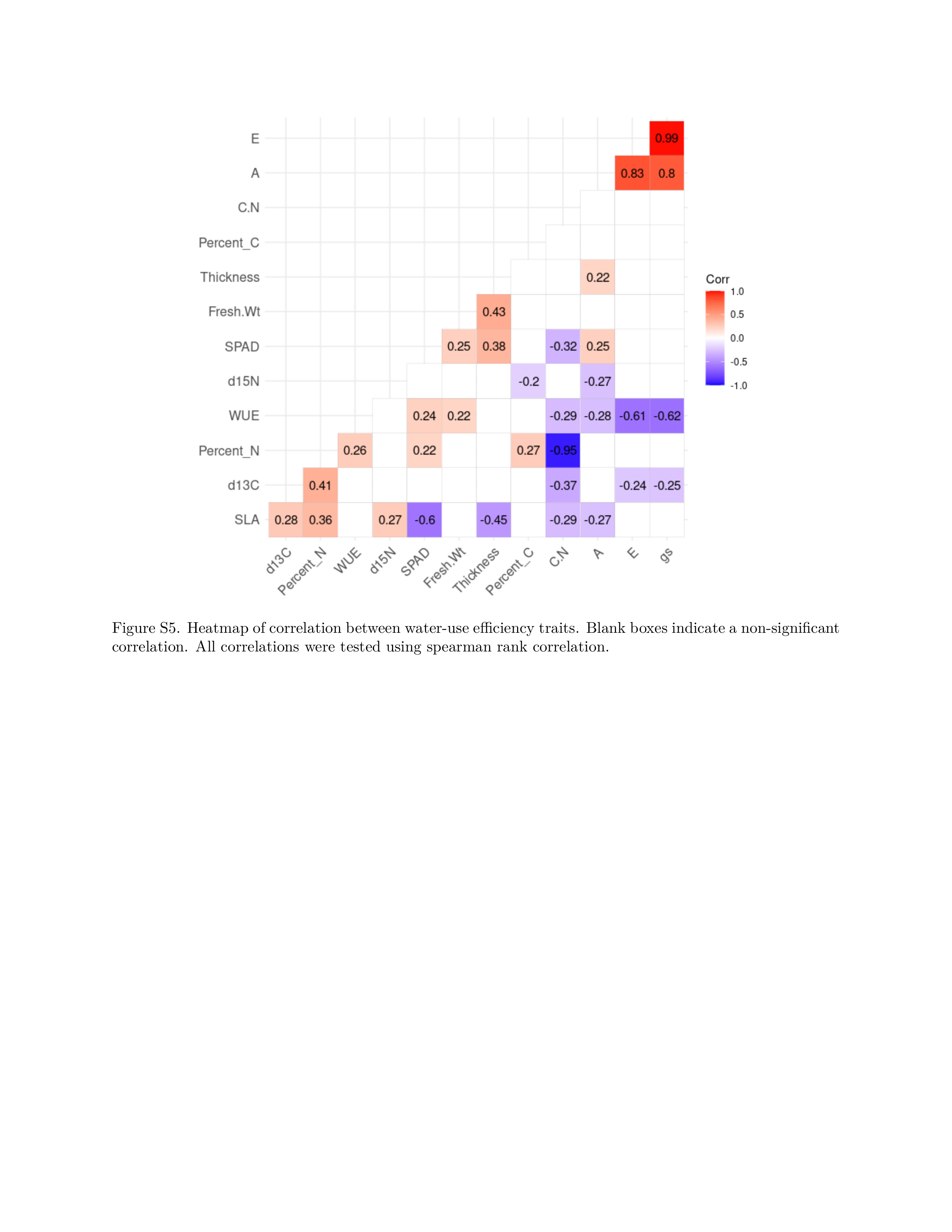

Supplement: Supplementary file 5 — Figure S5. Heatmap of correlation between water‐use efficiency traits. Blank boxes indicate a non‐significant correlation. All correlations were tested using spearman rank correlation. [file EVA-18-e70102-s001.png]

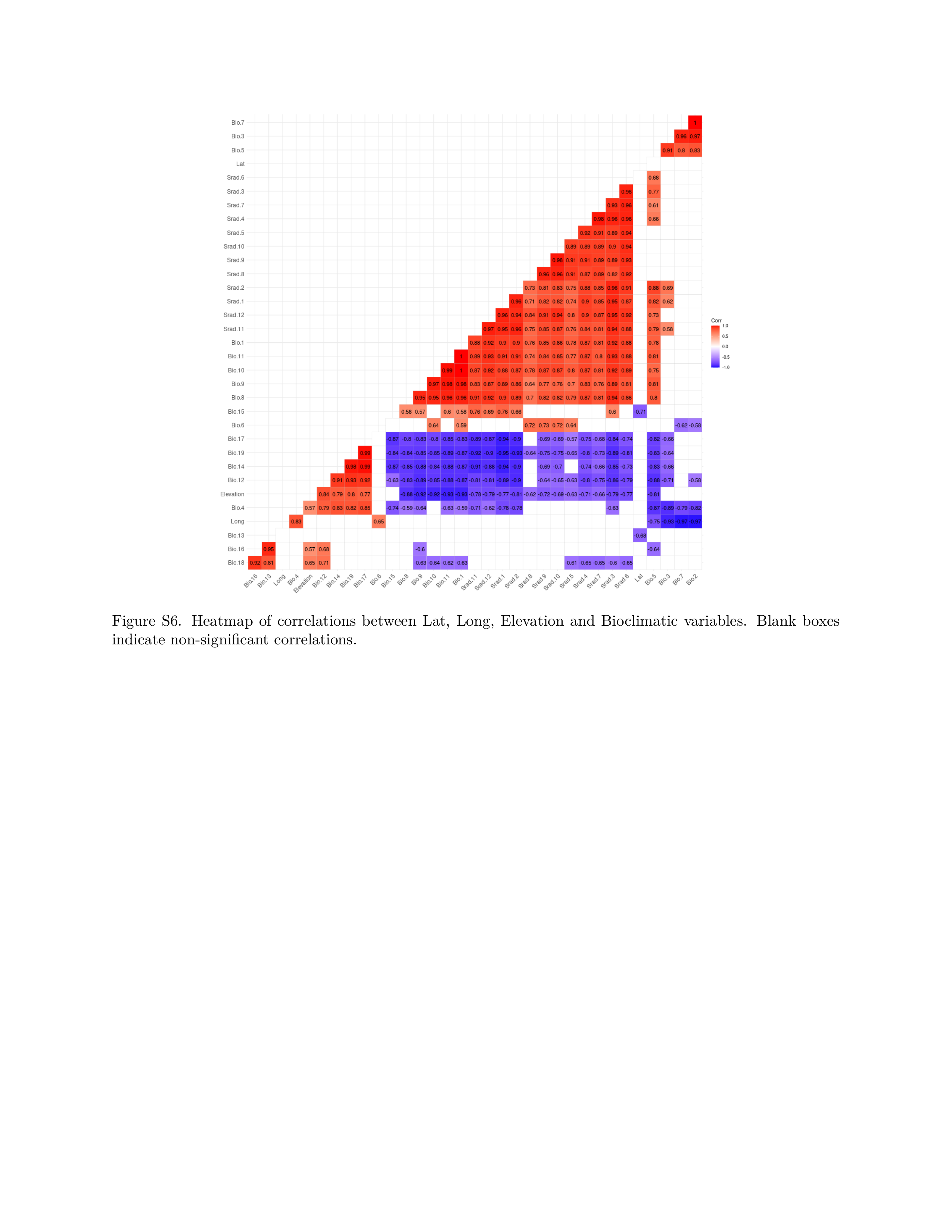

Supplement: Supplementary file 6 — Figure S6. Heatmap of correlations between Lat, Long, Elevation and Bioclimatic variables. Blank boxes indicate non‐significant correlations. [file EVA-18-e70102-s006.png]
